# Systemic Melioidosis With Ruptured Splenic Abscess

**DOI:** 10.7759/cureus.7956

**Published:** 2020-05-04

**Authors:** Sakthivel Chinnakkulam Kandhasamy, TP Elamurugan, Debasis Naik, Gorrepati Rohith, Vishnu Prasad Nelamangala Ramakrishnaiah

**Affiliations:** 1 Surgery, Jawaharlal Institute of Postgraduate Medical Education and Research, Puducherry, IND; 2 General Surgery, Jawaharlal Institute of Postgraduate Medical Education and Research, Puducherry, IND

**Keywords:** melioidosis, splenectomy, burkholderia pseudomallei, ruptured splenic abscess

## Abstract

Melioidosis is a severe systemic disease caused by the bacterium *Burkholderia pseudomallei*, commonly found in soil, ground water, and ponds of endemic regions. It is transmitted to humans via percutaneous inoculation while working in these areas without protective clothing and footwear giving rise to the disease which has a high case fatality rate. It has a wide range of clinical manifestations, varying from asymptomatic infection to localized abscess formation to fulminating disease with multiple organ involvement and even death. Currently, there are no known pathognomonic features or specific criteria which can lead to a confident diagnosis of melioidosis. The gold standard diagnostic test is culture sensitivity of blood, pus, or bodily fluids, which itself has a low sensitivity. Imaging findings are not specific and can mimic other bacterial infection. However, awareness of these radiographic manifestations in multiple organs can raise the possibility of diagnosis and lead to more early proper treatment and thereby lower the high mortality associated with this disease. We here present a rare case of systemic melioidosis with ruptured splenic abscess managed laboriously with antibiotics and splenectomy and wish to review the literature.

## Introduction

Melioidosis is a severe systemic infectious disease caused by the saprophyte *Burkholderia pseudomalle*i, which is commonly found in the soil, groundwater, rice paddies, and ponds throughout endemic regions. The disease is acquired through contact with contaminated soil or water by percutaneous inoculation, aerosol inhalation, or the ingestion of contaminated water or food. The case fatality rate ranges from 19% to 36% in endemic areas [1]. The risk factors being diabetes mellitus, tuberculosis, soil/water exposure history, chronic renal disease, excessive alcohol consumption, and steroid use [2]. It has a wide range of clinical manifestations, varying from asymptomatic infection to localized abscess formation to fulminating disease with multiple organ involvement and even death. Splenic abscess is rare presentation, even in endemic areas, with only a few cases reported. The disease is almost impossible to be diagnosed clinically. Hence, an awareness of the disease and knowledge of the various radiological detections may help in guiding and obtaining the appropriate diagnostic test to reach an early diagnosis. We here present a rare case of systemic melioidosis with ruptured splenic abscess managed laboriously with antibiotics and splenectomy and wish to review the literature.

## Case presentation

**Figure 1:**
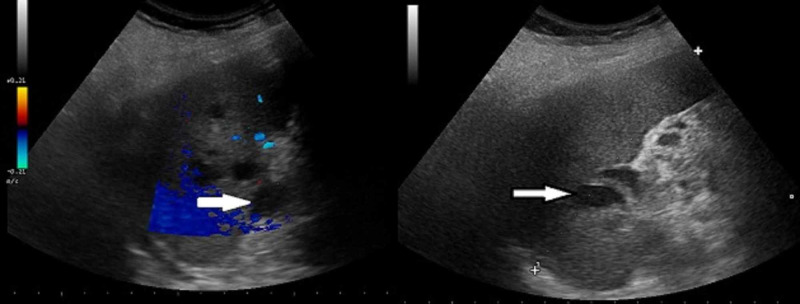
Ultrasonography showing multiple hypoechoic lesions in the spleen suggestive of splenic abscess (arrows).

**Figure 2:**
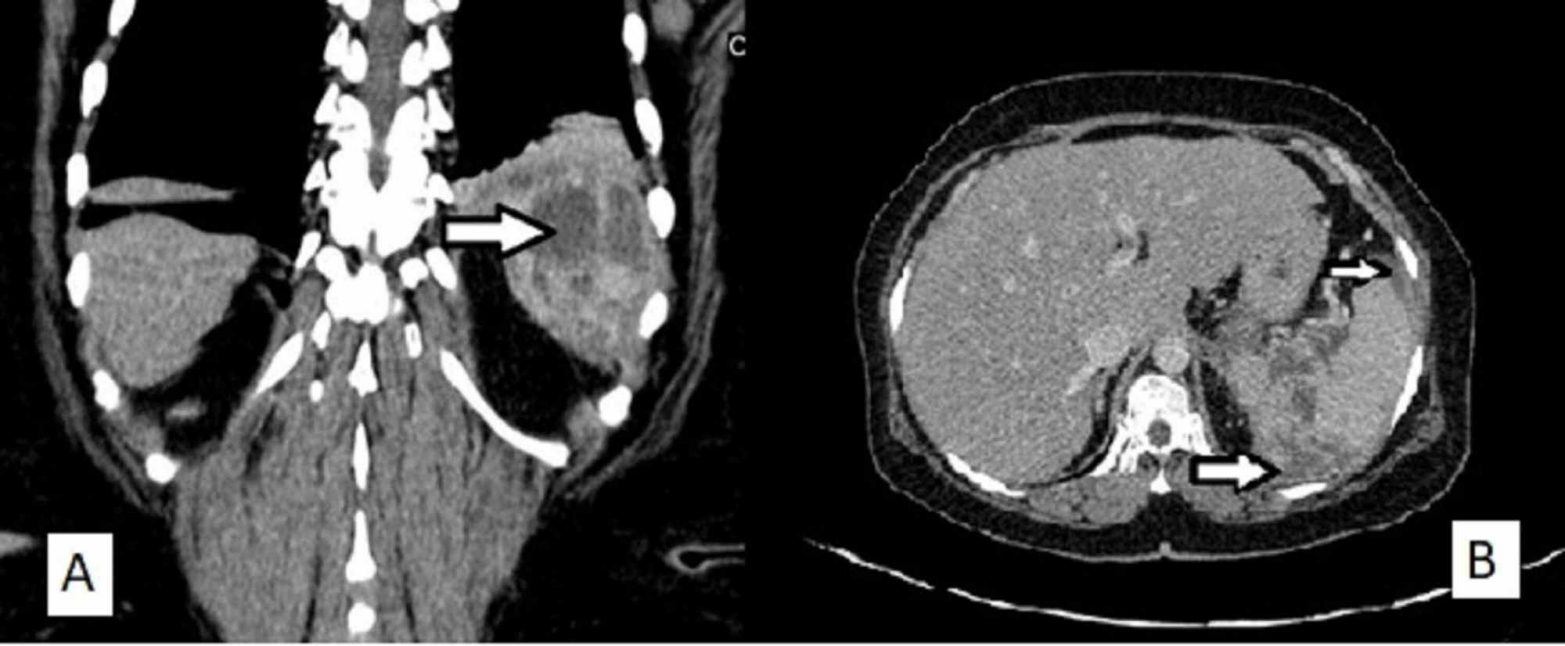
Contrast-enhanced computed tomography of abdomen. (A) Coronal section showing multiple abscesses in the spleen (arrow). (B) Transverse section showing multiple splenic abscesses and perisplenic collection (arrow).

A 55-year farmerette, a known diabetic on irregular treatment, presented with complaints of dull aching pain in the left upper abdomen for one month followed by high-grade fever associated with chills and rigors for 20 days. There was no associated nausea, vomiting, burning micturition, constipation or passage of loose stools, cough with expectoration, or difficulty in breathing. The patient was febrile and dehydrated. Vital signs were as follows: pulse, 112/min; blood pressure, 102/60 mmHg; chest, decreased breath sounds in the left side infrascapular region. Abdominal examination revealed tenderness in left hypochondrium, no guarding or rigidity, liver was not enlarged, and spleen was not palpable. Ultrasonography of the abdomen (Figure 1) and contrast-enhanced computed tomography of the abdomen (Figure 2) revealed a spleen of 11.2 cm size, with multiple heterogeneous hypoechoic ill-defined lesions, largest measuring 5.4 cm x 3.4 cm at the superior pole of the spleen, with no obvious vascularity suggestive of abscess. Another 4.9 cm x 1.1 cm hypoechoic area in the left subdiaphragmatic area adjacent to previous lesion suggestive of ruptured splenic abscess. There was minimal left-sided pleural effusion. Blood investigations were as follows: hemoglobin (Hb%) 10.6 g%, total leukocyte count (TLC) 8,030/mm^3 ^with 80% neutrophils, platelet count 2.06 lakhs/ mm^3^, random blood sugar (RBS) 358 mg/dL, and HbA1c 7.6 %.

**Figure 3:**
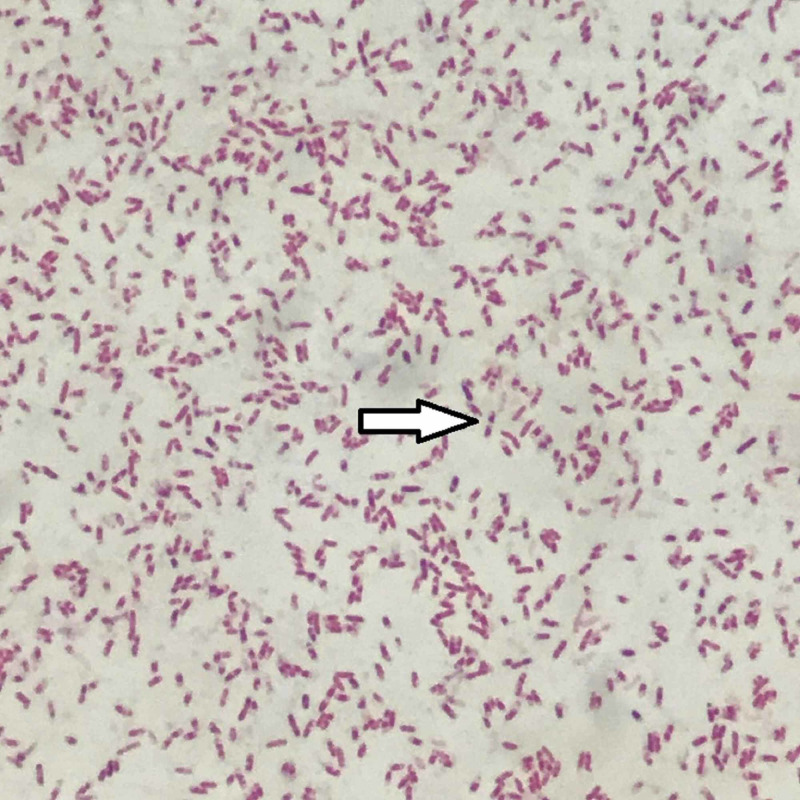
Gram stain of pus showing multiple gram-negative coccobacilli with safety pin appearance due to bipolar staining (arrow).

**Figure 4:**
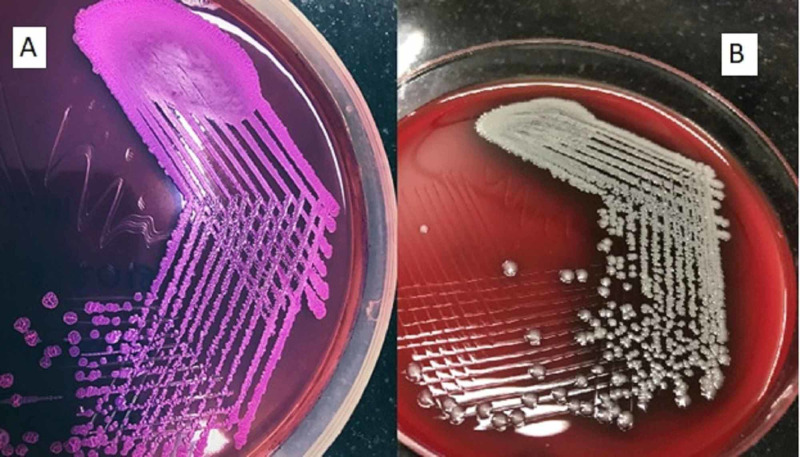
Bacterial culture plates showing growth of Burkholderia pseudomallei. (A) MacConkey agar showing growth of pale pink colonies. (B) Sheep blood agar showing the growth of large, silvery-white, round colonies with central umbonation.

The patient was resuscitated with intravenous (i.v.) fluids, blood sugars were brought under control with insulin infusion, and the patient was put on i.v. antibiotics ceftriaxone and metronidazole empirically. Her viral serologies were all negative. Echocardiography revealed normal left ventricular systolic function with no clots/vegetation. Blood culture came sterile after two days of incubation. Ultrasound-guided aspiration of pus from subdiaphragmatic collection was done on the next day of admission, the culture of which showed growth of *B. pseudomallei* (Figures 3, 4) sensitive to meropenem, which was started. Also aspiration was repeated multiple times in view of poor window for the placement of pig tail catheter. Despite this, the patient continued to have four to five episodes of high-grade fever (102-103^o^F) every day and by day 5 of meropenem she also developed multiple small abscesses of varying size all over her body especially on the extremities. In view of systemic presentation, drainage of all abscess cavities was planned and the patient was taken for laparotomy. Intraoperatively, there was around 100 ml of purulent left subdiaphragmatic collection, with around 5 cm x 4 cm ruptured abscess cavity at the superior pole of spleen. In view of multiple unresolved abscesses, splenectomy was done. This procedure was coupled with drainage of all superficial abscess over the extremities. Postoperatively, the patient continued to have episodes of fever but this time all low grade. Pus collected intraoperatively from spleen and superficial abscesses on culture showed growth for the same organism sensitive to ceftazidime, which was added to her treatment and continued for 14 days along with meropenem. The wounds following drainage of superficial abscesses started healing and her fever subsided. On sixth postoperative day, the patient develop dyspnea, which on evaluation revealed left moderate pleural effusion with left lower lobe atelectasis. Around 750 ml of straw colored fluid was aspirated from left pleural cavity following which the patient improved and was discharged on oral cotrimoxazole and doxycycline after vaccination against pneumococcus, meningococcus, and *Haemophilus influenzae*. Again after five days she came back with complaints of fever with multiple abscesses over the body. Ultrasound revealed 5.5 cm x 2 cm thick collection in the left subdiaphragmatic region with no window for percutaneous catheter drainage. The patient underwent relaparotomy with lavage and drainage of all superficial abscess. The patient was again started on meropenem and ceftazidime and continued for 14 days and discharged after her condition improved on oral doxycycline and cotrimoxazole. 

 

## Discussion

Melioidosis is caused by the gram-negative bacteria, *B. pseudomallei*. First recognized in Rangoon, Burma in April 1911 by Alfred Whitemore and C. S. Krishnaswami, it was called the Whitmore’s disease [3]. In 1932, Stanton and Fletcher published their monograph on the disease, which they named melioidosis (Greek, *melis*, a distemper of asses, *eidos*). This was because the disease clinically and pathophysiologically resembled glanders, a chronic debilitating disease of equines due to *Pseudomonas mallei*. The causative organism was earlier called *Pseudomonas pseudomallei* [4]. In 1992, Walter Burkholder named a new genus *Burkholderia* and moved seven species of *Pseudomonas* including *pseudomallei* into it [5].

*B. pseudomallei* is present in soil and surface water in areas where melioidosis is endemic, and most cases are thought to result from bacterial inoculation based on the observations that people at high risk of melioidosis such as agricultural workers in Thailand and indigenous people in Australia are regularly exposed to soil and water without protective clothing and may suffer repeated minor injuries [6-8]. The role of other routes of infection like inhalation and ingestion is uncertain.

Melioidosis is endemic in South and East Asia, Northern Australia, the Indian subcontinent, and areas of South America [9-11]. Northeast Thailand is a hotspot for this infection, with an annual incidence of 21.0 per 100,000 population and a crude mortality rate of 40%. This rate is comparable to that for deaths from tuberculosis in this region, where melioidosis is the third most common cause of death from infectious diseases [12]. Visitors to areas where melioidosis is endemic are also at risk of acquiring this infection.

Clinically meliodosis can present with fever, septicemia, or localized abscess. Almost every organ can be affected with the most common being lungs, skin, and subcutaneous tissue, and visceral organs such as the spleen and liver. Rare sites of involvement include central nervous system, bone and joints, and cardiac and vascular systems. Although lymph nodes can be involved by *B. pseudomallei*, suppurative lymphadenitis is a rare presentation [13].

Currently there are no known pathognomonic features or specific criteria which can lead to a confident diagnosis of melioidosis. When melioidosis is suspected, the gold standard diagnostic test is culture of blood, pus, or bodily secretions, but even this test has a sensitivity of only 60% [14]. Besides cultures, other tests like the indirect hemagglutination antibody (IHA) test or immunofluorescent assays (IFA) for immunoglobulin M or G (IgM, IgG), which have sensitivities of 76% and 73% and specificities of 91% and 99%, respectively, have been used in the diagnosis [15,16]. Polymerase chain reaction to detect *B. pseudomallei* has also been used to aid in the diagnosis. In our case, the diagnosis of melioidosis was not made until the culture report of pus was obtained. Radiological features that may help in diagnosis are multiple, small, and discrete, splenic lesions varying in size from 0.5 to 1.5 cm, single or multiple multiloculated lesions, subcapsular collections with or without perisplenic extension [17]. Single or multiple splenic abscesses are more commonly found in melioidosis than in other infections. Concurrent spleen and liver abscess are more likely to be associated with melioidosis than with infections caused by other organisms.

Based on randomized and semirandomized controlled clinical trials of drug regimens, effective treatments for severe acute infection include i.v. ceftazidime (with or without trimethoprim-sulfamethoxazole), meropenem, amoxicillin-clavulanic acid, imipenem, and cefoperazone-sulbactam for several weeks, followed by oral treatment with trimethoprim-sulfamethoxazole and doxycycline for five months [18]. Despite this, the mortality remains high around 40%. Doxycycline can be used to treat localized melioidosis, whereas combination with other antibiotics is required to alleviate systemic disease [2]. Melioidosis can become chronic with formation of abscesses or remain subclinical for many years, probably due to the ability of the microorganism to survive within phagocytes with the risk of reactivation precipitated by immunosuppression. Chronic melioidosis is treated with i.v. ceftazidime for at least two weeks, followed by oral therapeutics given up to three months for the complete abolition of infection [19].

Necessary preventive strategies should be employed in high-risk populations to prevent contacting this severe systemic disease, such as avoiding direct exposure of contaminated clay soil and standing water in prevalent areas. In addition, clinicians examining travelers with severe pneumonia or septicemia returning from the subtropics or tropics should consider the differential diagnosis of acute melioidosis.

## Conclusions

Melioidosis is an important public health bacterial infection, with a wide variety of clinical manifestations and can affect many organs. The lung being the most commonly infected followed by the spleen and liver, with the most frequent presentation being fever with single or multiple abscess. Imaging findings are not-specific and mimic other bacterial infection. However, awareness of these radiographic manifestations in multiple organs can raise the possibility of diagnosis and lead to more early and aggressive treatment with surgical drainage and antibiotics for several weeks and thereby lower the high mortality associated with this disease.
